# Liquid-infused interfacial floatable porous membrane as movable gate for ultrafast immiscible oil/water separation

**DOI:** 10.1038/s41598-023-40262-x

**Published:** 2024-01-02

**Authors:** Jianlin Yang, Xin Yang, Tianlu Yu, Zhecun Wang

**Affiliations:** 1 College of Materials Science and Engineering, Liaoning Technical University, Fuxin, 123000 China; 2 School of Civil Engineering, Liaoning Technical University, Fuxin, 123000 China

**Keywords:** Pollution remediation, Wetting

## Abstract

Liquid separation methods are widely used in industrial and everyday applications, however, their applicability is often constrained by low efficiency, membrane fouling, and poor energy efficiency. Herein, a conceptually novel liquid-infused interfacial floatable porous membrane (LIIFPM) system for high-performance oil/water separation is proposed. The system functions by allowing a liquid to wet and fill a superamphiphilic porous membrane, thereby creating a stable liquid-infused interface that floats at the oil/water interface and prevents the passage of immiscible liquids. The lower-layer liquid can outflow directly, while the flow of the upper-layer liquid is stopped by the membrane. Remarkably, the efficiency of the LIIFPM system is independent of the membrane pore size, enabling ultrafast immiscible oil/water separation in an energy-saving and antifouling manner.

## Introduction

Liquid separation methods are widely used in everyday life and industrial activities, such as oily wastewater treatment. Driven by the need for high throughput and energy efficiency, solid porous separation membranes have gained considerable attention for these applications^1–3^. Solid porous membranes that display contrasting wettability toward oil and water^4–7^, such as superhydrophilic/underwater superoleophobic^8–13^ or superhydrophobic/superoleophilic membranes^14–18^, are preferred for the continuous separation of immiscible oil/water mixtures, as they offer high selectivity and efficiency^19–24^. However, solid porous membranes have several constraints, including fouling issues, physical damage, poor stability, and lack of self-healing capabilities, which severely limit their applications.

To address the aforementioned problems, liquid-infused porous membranes have been developed, in which the lubricant liquid within the membrane acts as a reconfigurable barrier^25–30^. These nature-inspired liquid-based membranes demonstrate exceptional multiphase liquid separations. Nevertheless, they suffer from several issues. For example, perfluorinated fluids are currently required for the liquid, and the pressure must be precisely adjusted to control the gating threshold^31–33^. Our group previously prepared a liquid-infused patterned porous membrane^34^ and a liquid-based Janus porous membrane co-infused with water and oil^35^. These membranes showed excellent interfacial floatability for high-performance liquid separations; however, they required a multi-step fabrication process that hindered their practical applicability. Thus, there is a pressing need to develop simple liquid-based membrane systems for high-performance liquid separation.

A notable constraint of conventional filtration membranes is the fouling problem. The membrane pores eventually become clogged by micrometer-scale pollutant particles and viruses, which ultimately obstruct permeation. A microstructural sponge design has been proposed to address the problem of pore blockage^36–38^. In addition, immiscible oil/water separation is conventionally realized using a fixed porous membrane, through which the liquid travels under the action of gravity. In such systems, permeation theory shows that the flux is directly proportional to the pore size and inversely proportional to membrane thickness^39–45^. Consequently, for ultrafast separation, the membrane should have both an ultrathin separation layer and a large pore size. However, maintaining membrane integrity while enduring the pressure of the filtration liquid necessitates a certain membrane thickness, making the optimization of these two features in the same membrane challenging.

In this study, we introduce a conceptually different immiscible oil/water separation system that uses a liquid-infused interfacial floatable porous membrane (LIIFPM). This novel system employs a readily accessible liquid-infused superamphiphilic porous membrane as a movable barrier that floats at the oil/water interface. It allows the lower-layer liquid to outflow directly and self-gates the upper-layer liquid. Importantly, the efficiency of the LIIFPM system is independent of the membrane pore size, which deviates from conventional fixed-membrane systems. This innovative system facilitates ultrafast separation and holds significant promise for immiscible oil/water separation.

## Materials and methods

### Materials

Petroleum ether, cyclohexane, heptane, *n*-hexane, carbon tetrachloride (CCl_4_), oil red, poly(vinylidene fluoride-*co*-hexafluoro propylene) (PVDF-HFP), reactive red, and methyl blue were obtained from Aladdin Co., Ltd (China). A cellulose acetate (CA) membrane (pore size: 0.22 μm) was procured from EMD Millipore Corporation (USA), and filter paper was obtained from Beijing North Dawn Membrane Separation Technology Corporation (China).

### Oil/water separation

For oil/water separation using the LIIFPM technology, a lower-layer liquid-infused membrane (radius: 3 cm) was placed in a glass tube (pore size: 1.0 cm) containing 200 mL of an immiscible oil/water mixture (1:1 v/v). Upon removal of the rubber plug at the bottom of the tube, the lower-layer liquid swiftly flows from the tube, while the upper-layer liquid is stopped from leaving the tube by the lower-layer liquid-infused membrane.

### Surface and interfacial tension experiments

The surface and interfacial tensions of different liquids at room temperature were measured using a surface tension coefficient instrument (Sigma 702ET, Biolin Scientific, Sweden). Table 1 lists the surface and interfacial tension values.

**Table 1:** The surface tension and interfacial tension of different liquids.

	Interfacial tension (mN/m)	Surface tension (mN/m)
Water	–	73.89
Petroleum ether	–	27.64
*n*-hexane	–	27.54
Cyclohexane	–	28.42
CCl_4_	–	45.8
Water/petroleum ether	62.27	–
Water/*n*-hexane	70.58	–
Water/cyclohexane	65.18	–
Water/CCl_4_	43.18	–

### Absorption capacity

The filter paper and CA membrane were immersed in different solvents for 5 min. Immediately afterward, the weights of the membranes and absorbed solvents were measured. The absorption capacity was calculated as *W*_1_/*W*_0_, where *W*_1_ is the weight of the absorbed solvent and *W*_0_ is the weight of the membrane.

### Characterization

The contact angles of different liquids were measured using 5 μL droplets (Drop Shape Analysis DSA10, Krüss Gmbh, Germany). The morphology was determined by scanning electron microscopy (SEM; Philips XL30 ESEM FEG). The Fourier-transform infrared (FT-IR) spectra were acquired using a Bruker Vertex 70 spectrometer.

## Results and discussion

### Separation mechanism

**Figure 1:**
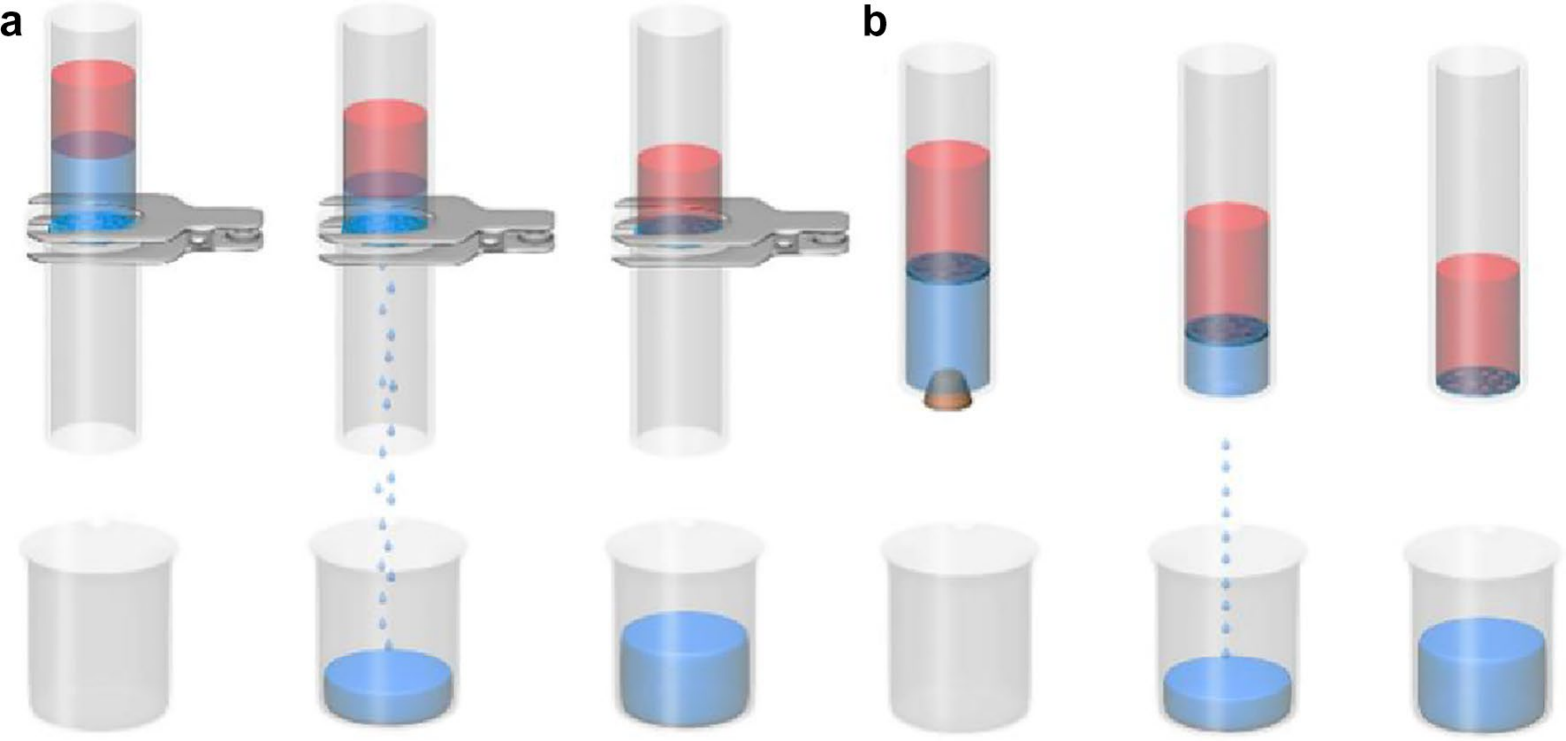
The schematic of (**a**) traditional oil/water filtration, and (**b**) LIIFPM system for oil/water separation (The separation is performed with a glass tube, and to prevent the membrane from falling off the bottom of the glass tube, there is a hole underneath that is smaller in size than the inner diameter of the glass tube, which can be sealed with a rubber plug to control the flow of liquid in and out through the hole).

Figure 1 compares the traditional and LIIFPM techniques for the separation of immiscible oil/water mixtures. In traditional filtration system, the porous membrane is affixed to a device and operates without movement (Fig. 1a). Although fixed porous membranes can separate immiscible oil/water mixtures, they are highly susceptible to pore-clogging. Figure 1b illustrates the separation mechanism using the LIIFPM technique. Here, the lower-layer liquid-infused porous membrane floats at the oil/water interface. As the lower-layer liquid flows out the tube, the porous membrane impedes the flow of the upper-layer immiscible liquid, thereby accomplishing comprehensive separation. Since the filtrate does not pass through the porous membrane, the separation flux of the system is extremely high, enabling high-efficiency separation via gravity. Consequently, this method has significant potential for use in wastewater treatment.

### Three criteria for the LIIFPM system

The conceptualization of the LIIFPM-based oil/water separation technique hinges on three criteria^27,46^: (1) the porous membrane must be completely filled with the lower-layer liquid, without any trapped air, thereby forming a defect-free liquid film; (2) the lower-layer liquid should wet the porous membrane preferentially and reject the immiscible upper-layer liquid. This ensures that the infused liquid is not displaced by the other liquid; and (3) the lower-layer liquid-infused porous membrane must remain afloat at the oil/water interface throughout the separation process.

### The first criterion

The lower-layer liquid-infused porous membrane gates the upper-layer immiscible liquid, allowing perfect separation once the lower-layer liquid has drained out, as depicted in Fig. 1b. If the porous membrane is not entirely filled with the lower-layer liquid and contains wettable defects, it could be infiltrated by the upper-layer liquid upon contact, resulting in the outflow of the upper-layer liquid as well as the lower-level liquid. Therefore, to achieve separation, a pivotal criterion of our design is that the porous membrane must be perfectly infused by the lower-layer liquid, creating a defect-free liquid surface.

**Figure 2:**
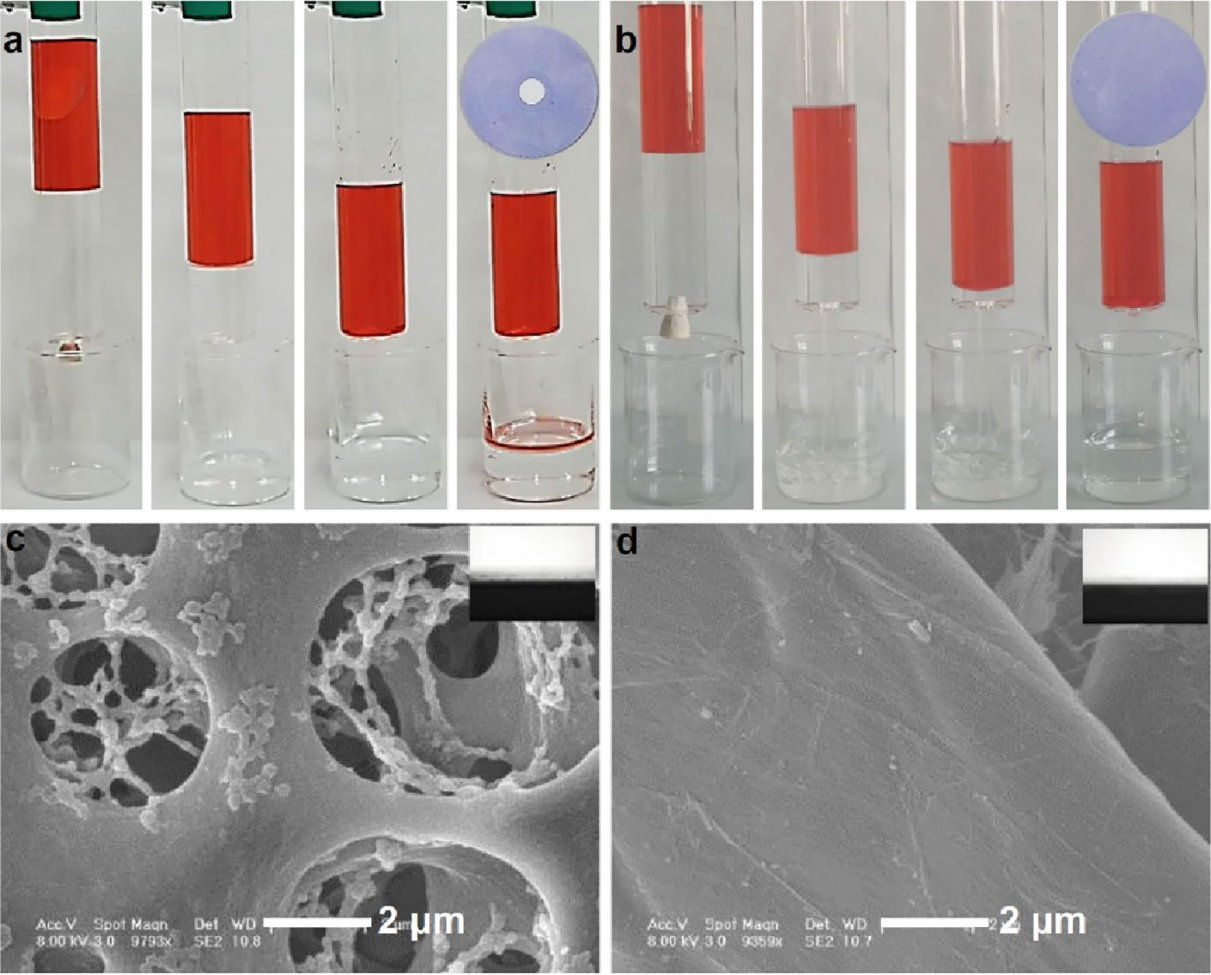
(**a**) The hydrophobic solution (1% PVDF-HFP in DMF solution) treated CA (pore size: 0.22 μm) cannot be fully wetted by water (dyed by methyl blue) with a circle defect (insert picture), and the solvent (petroleum ether/water mixture) would be leakage through the defect CA, (**b**) the original CA can be fully wetted by water (dyed by methyl blue) without defect (insert picture), and it could separate the water from the mixture perfectly. The morphology and wettability (insert picture) of CA (**c**) and filter paper (**d**).

To underscore the significance of this criterion, we performed petroleum ether/water separation experiments using both a wettable defect-free CA membrane (original) and a wettable defect-containing CA membrane (control). The control membrane was prepared by partial hydrophobic modification of the original CA membrane using PVDF-HFP^14^, rendering it incapable of complete infusion by the lower-layer liquid (water, dyed by methyl blue). This resulted in the formation of a small circular air-pocket (diameter: 1.5 cm) (Fig. 2a, inset). Conversely, the original CA membrane was completely infused by the lower-layer liquid (water, dyed by methyl blue), thus forming a wettable defect-free porous membrane (Fig. 2b, inset). The control membrane failed to separate the oil/water mixture, while the original CA membrane effectively separated the oil/water mixture, underscoring the importance of this criterion (Fig. 2a, b).

Thus, the first criterion is satisfied by using a porous membrane, akin to a reservoir, that is fully wettable by the lower-level liquid. This ensures complete wetting and filling of the liquid into the membrane, thereby forming a defect-free film. To facilitate the filling of the porous membrane by oil or water, the liquid must readily displace the air in the porous membrane upon contact. In this context, we only need to consider the water filling principle, which is a sufficient and necessary condition for oil filling, given that oil has a lower surface tension (smaller contact angle) than water and thus fills the porous membrane more easily.

CA and filter paper membranes both have a low water contact angle. Therefore, they can both form defect-free water films (Fig. 2c, d). As such, these porous membranes can satisfy the first criterion of forming a defect-free water or oil film within the porous membrane.

### The second criterion

**Figure 3:**
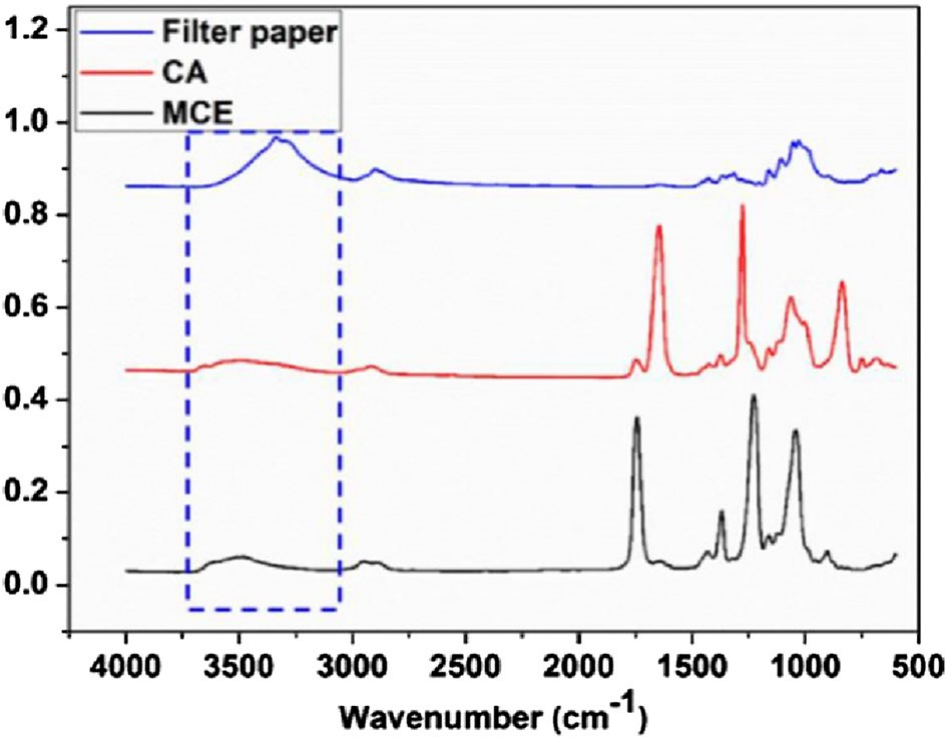
The FTIR of different porous membranes.

**Figure 4:**
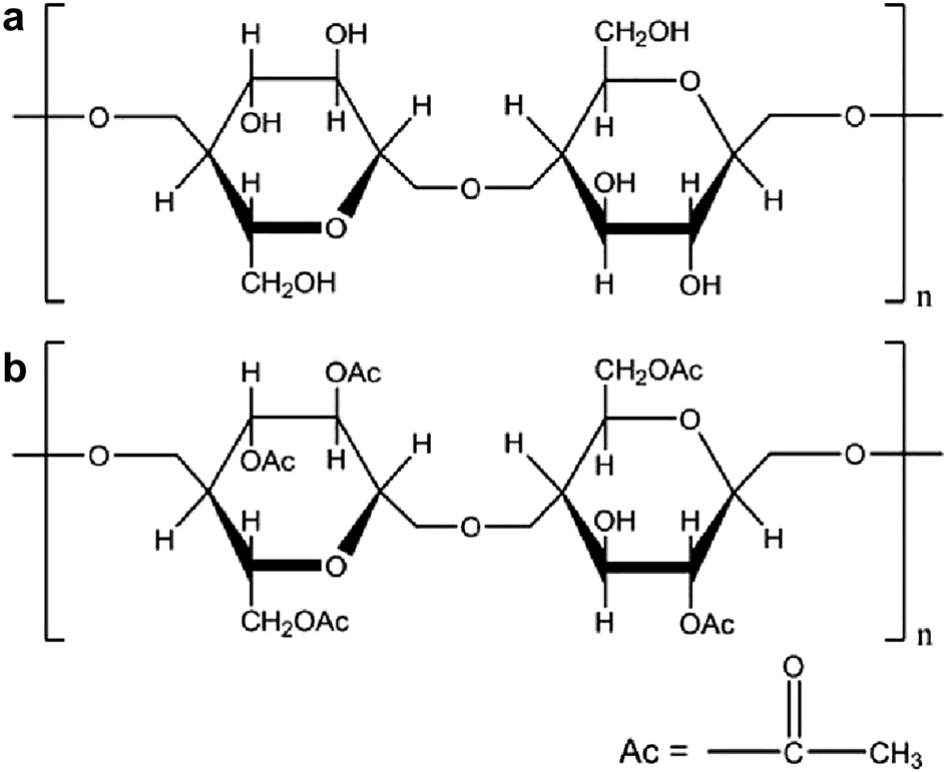
The molecular structures of different materials, (**a**) filter paper, (**b**) CA.

The second criterion plays a pivotal role in achieving perfect separation. The membrane must repel the introduced liquid, thereby ensuring the liquid filling the porous membrane remains undisturbed. To illustrate the significance of this criterion, we used filter paper as a counter-example for oil/water separation. The filter paper has an exceptional binding affinity toward water^47^. The FTIR spectra and molecular structure indicate that the filter paper contains numerous hydrophilic hydroxyl groups (Figs. 3 and 4), facilitating strong interactions with water. Meanwhile, CA contains both hydrophilic hydroxyl groups and hydrophobic cycloparaffin domains (Fig. 4b), which bestow it with a distinctive amphiphilic nature.

**Figure 5:**
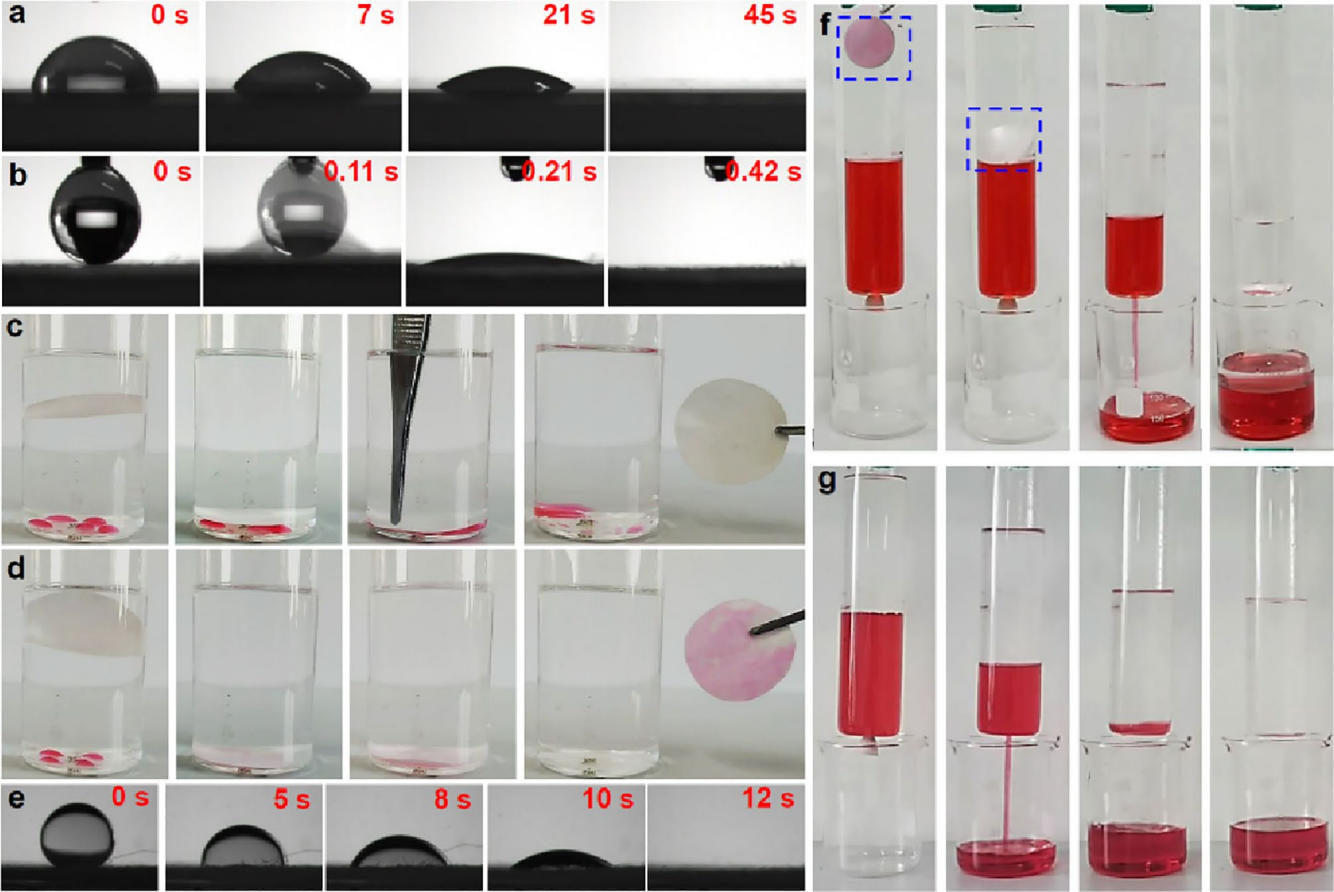
The water (10 μL) contact angle of CA (**a**) and filter paper (**b**) in air, (**c**) The petroleum ether-infused CA membrane cannot absorb the water (dyed by reactive red) from petroleum ether, (**d**) The petroleum ether-infused filter paper can absorb the water (dyed by reactive red) from petroleum ether, (**e**) Under-oil (petroleum ether) environment, the oil in filter paper is displaced by a drop of water (the dynamic change of water contact angle under petroleum ether), (**f**) The CCl_4_-infused filter paper, which is de-wetted by water, cannot separate the water/CCl_4_ mixture, (**g**) The CCl_4_-infused CA can separate the water/CCl_4_ mixture.

The time required for water (10 μL) to fully spread into the porous CA membrane was 11 s (Fig. 5a). In contrast, it took only 0.6 s for water (10 μL) to completely spread into the dry filter paper (Fig. 5b). A petroleum ether-infused CA membrane was unable to absorb water from a petroleum ether/water mixture (Fig. 5c). In contrast, petroleum ether-infused filter paper was able to absorb water from a petroleum ether/water mixture (Fig. 5d). This indicates that, because the interactions between the filter paper and oil molecules are dominated by van der Waals interactions, the oil-infused filter paper can be de-wetted at the oil/water interface owing to the strong hydration of water (hydrogen bonding interactions). As a result, the oil was displaced by water (Fig. 5e). Similarly, when CCl_4_-infused filter paper was used to separate a water/CCl_4_ mixture, the CCl_4_ (dyed by oil red) in the filter paper was displaced by water immediately upon contact owing to the rapid and complete de-wetting of the filter paper, causing the water to leak through the membrane. Hence, the water/CCl_4_ mixture could not be separated by the CCl_4_-infused filter paper (Fig. 5f). This outcome starkly contrasts with that using a CCl_4_-infused CA membrane, which successfully separated CCl_4_ from a water/CCl_4_ mixture (Fig. 5g). These results underscore the importance of the second criterion.

**Figure 6:**
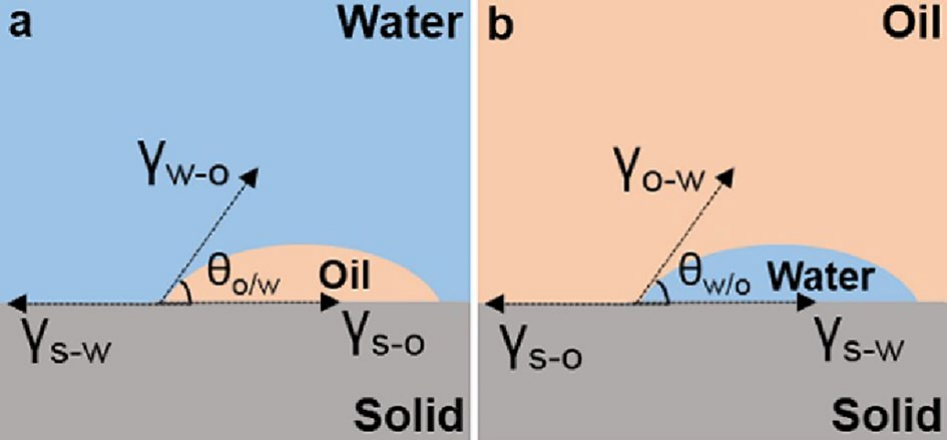
Schematic of an oil drop on the surface of water-filled porous membrane in water (**a**) and a water drop on the surface of oil-filled porous membrane in oil (**b**).

Repellency is a critical factor for separation, rendering the fulfilment of the second criterion necessary. To satisfy the second criterion in underwater environments, oil droplets must remain stable on the surface of the water-infused membrane. Thermodynamic equilibrium must be achieved among the oil phase, solid phase, and water phase. According to Young’s equation (Fig. 6a),

1 $$\gamma_{{{\text{s}}/{\text{w}}}} = \gamma_{{{\text{s}}/{\text{o}}}} + \gamma_{{{\text{w}}/{\text{o}}}} {\text{cos}}\theta_{{{\text{o}}/{\text{w}}}}$$

where *γ*_s/o_, *γ*_s/w_, and *γ*_w/o_ are the solid/oil, solid/water, and water/oil interfacial tensions, respectively, and *θ*_o/w_ is the oil contact angle in water. The Gibbs free energy (Δ*G*) for replacement of the solid/water interface with a solid/oil interface can be expressed as^27^

2 $$\Delta G_{1} = R\left( {\gamma_{w} \cos \theta_{w} - \gamma_{o} \cos \theta_{o} } \right) - \gamma_{w/o}$$

where *γ*_w_ and *γ*_o_ are the water surface tension and oil surface tension, respectively, *θ*_o_ and *θ*_w_ are the apparent oil contact angle in air and apparent water contact angle in air, respectively, and *R* is the roughness factor of solid. For replacement of the filled water by oil, Δ*G*_1_ < 0; for repellence of oil by the filled water, Δ*G*_1_ > 0.

Similarly, in underoil environments, water droplets must remain stable on the surface of the oil-infused membrane. According to Young’s equation (Fig. 6b),

3 $$\gamma_{{{\text{s}}/{\text{o}}}} = \gamma_{{{\text{s}}/{\text{w}}}} + \gamma_{{{\text{w}}/{\text{o}}}} {\text{cos}}\theta_{{{\text{w}}/{\text{o}}}}$$

where *θ*_w/o_ is the water contact angle in oil. Consequently, the Gibbs free energy for replacement of the solid/oil interface with a solid/water interface can be expressed as

4 $$\Delta G_{2} = R\left( {\gamma_{o} \cos \theta_{o} - \gamma_{w} \cos \theta_{w} } \right) - \gamma_{o} + \gamma_{w}$$

For replacement of the filled oil by water, Δ*G*_2_ < 0; for repellence of water by the filled oil, Δ*G*_2_ > 0.

To adhere to the second criterion, which calls for the creation of a stable water or oil film that cannot be replaced by immiscible oil or water, we tailored the chemical and physical properties of the porous membranes to enable them to work in combination with water to repel oils. We compared the total interfacial energies of porous membranes that were preferentially wetted by water and that either repelled or absorbed a drop of immiscible oil in an underwater environment. To ensure that the water-infused porous membranes could repel the immiscible oil with sufficient stability, it is necessary for Δ*G* to be greater than 0.

In an underwater environment, Δ*G*_1_ was greater than 0 for both the water-infused filter paper and water-infused CA membrane (Table 2). As a result, both membranes can form stable oil-repellent solid/water interfaces against a variety of immiscible oils, such as petroleum ether, *n*-hexane, cyclohexane, and CCl_4_.

**Table 2:** In underwater environment, the instantaneous apparent contact angles of different liquids for different membranes, and the Gibbs free energy (Δ*G*_1_) for different systems.

Solid	Oil	*R*	*γ*_w_	*γ*_o_	*γ*_w/o_	*θ*_w_	*θ*_o_	Δ*G*_1_
Filter paper	petroleum ether	2	73.89	27.64	62.27	0	0	30.23
Filter paper	*n*-hexane	2	73.89	27.54	70.58	0	0	22.12
Filter paper	cyclohexane	2	73.89	28.42	65.18	0	0	25.76
Filter paper	CCl_4_	2	73.89	45.80	43.18	0	0	13.0
CA	petroleum ether	2	73.89	27.64	62.27	49.52	18.61	18.45
CA	*n*-hexane	2	73.89	27.54	70.58	49.52	20.28	26.18
CA	cyclohexane	2	73.89	28.42	65.18	49.52	19.78	22.60
CA	CCl_4_	2	73.89	45.80	43.18	49.52	18.19	34.14

In a similar vein, for a porous membrane that is preferentially wetted by oil. In this context, to ensure the filled oil can repel water, Δ*G*_2_ must be greater than 0. All the oil-infused filter papers (with different oils) had Δ*G*_2_ values of below 0 in underoil environments, indicating that the oils in the oil-infused filter papers could be replaced by water. That is, the filter paper has stronger affinity toward water than oil. This is in good agreement with the experimental observations. In contrast, the oil-infused CA membranes had Δ*G*_2_ values of above 0 in underoil environments (Table 3), indicating that these porous membranes possess extraordinary water repellency. Consequently, while both the water-infused filter paper and water-infused CA membranes can separate water from low-density oil, only the oil-infused CA membrane can separate high-density oil from water.

**Table 3:** In underoil environment, the instantaneous apparent contact angles of different liquids for different membranes, and the Gibbs free energy (Δ*G*_2_) for different systems.

Solid	Oil	*R*	*γ*_w_	*γ*_o_	*θ*_w_	*θ*_o_	Δ*G*_2_
Filter paper	Petroleum ether	2	73.89	27.64	0	0	− 46.25
Filter paper	*n*-hexane	2	73.89	27.54	0	0	− 46.35
Filter paper	Cyclohexane	2	73.89	28.42	0	0	− 45.47
Filter paper	CCl_4_	2	73.89	45.80	0	0	− 28.09
CA	petroleum ether	2	73.89	27.64	49.52	18.61	2.43
CA	*n*-hexane	2	73.89	27.54	49.52	20.28	1.96
CA	Cyclohexane	2	73.89	28.42	49.52	19.78	2.90
CA	CCl_4_	2	73.89	45.80	49.52	18.19	19.05

### The third criterion

**Figure 7:**
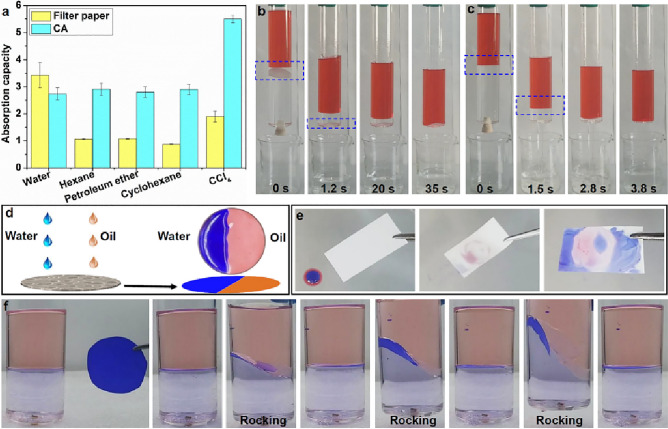
(**a**) The absorption capacity of the filter paper and CA membrane. (**b**) The water-infused filter paper sinks at the bottom (insert picture) can separate the water from petroleum ether slowly, (**c**) The water-infused CA floating at the petroleum ether–water interface (insert picture) enables to separate the water from petroleum ether rapidly, (**d**) Schematic description of the spreading of water and oil on CA membrane, and the water and oil can coexist at the same membrane, (**e**) A pierce of CA can absorb the water (10 μL) and water–immiscible oil (petroleum ether, 10 μL) at the same time forming the patterned morphology, (**f**) The water-infused CA membrane can float at the petroleum ether–water interface with stability after several times of rocking.

The third criterion serves as a critical condition for ultrafast separation. To verify the importance of this criterion, petroleum ether/water separation experiments were conducted using the water-infused filter paper and water-infused CA membrane at varying locations. Owing to its remarkable water affinity, the superhydrophilic filter paper has an excellent water absorption capacity; it can absorb approximately three times more water than it can any of the oils (Fig. 7a). Consequently, the water-infused filter paper easily sinks in water (lower-layer liquid), akin to traditional filtration processes, thereby facilitating slow oil/water separation (Fig. 7b). Conversely, the water-infused CA membrane floats stably at the oil/water interface for ultrafast oil/water separation (Fig. 7c).

To adhere to the third criterion, we selected a superamphiphilic porous CA membrane, which has a contact angle of 0° for both water and typical immiscible organic liquids. This ensures the membrane floats at the interface between these liquids. The CA membrane rapidly absorbs both water-immiscible oil (petroleum ether) and water simultaneously (Fig. 7d and e), demonstrating its superamphiphilic property.

Notably, the CA membrane has almost the same absorption capacity for both water and oils (Fig. 7a). If the water-infused CA membrane contacts the water-immiscible oil, the hydrophobic domain stretches to contact the oil, generating weak van der Waals forces between the membrane and oil molecules. Therefore, due to the superamphiphilic property, the water-infused CA membrane naturally floats at the petroleum ether/water interface (Fig. 7f).

**Figure 8:**
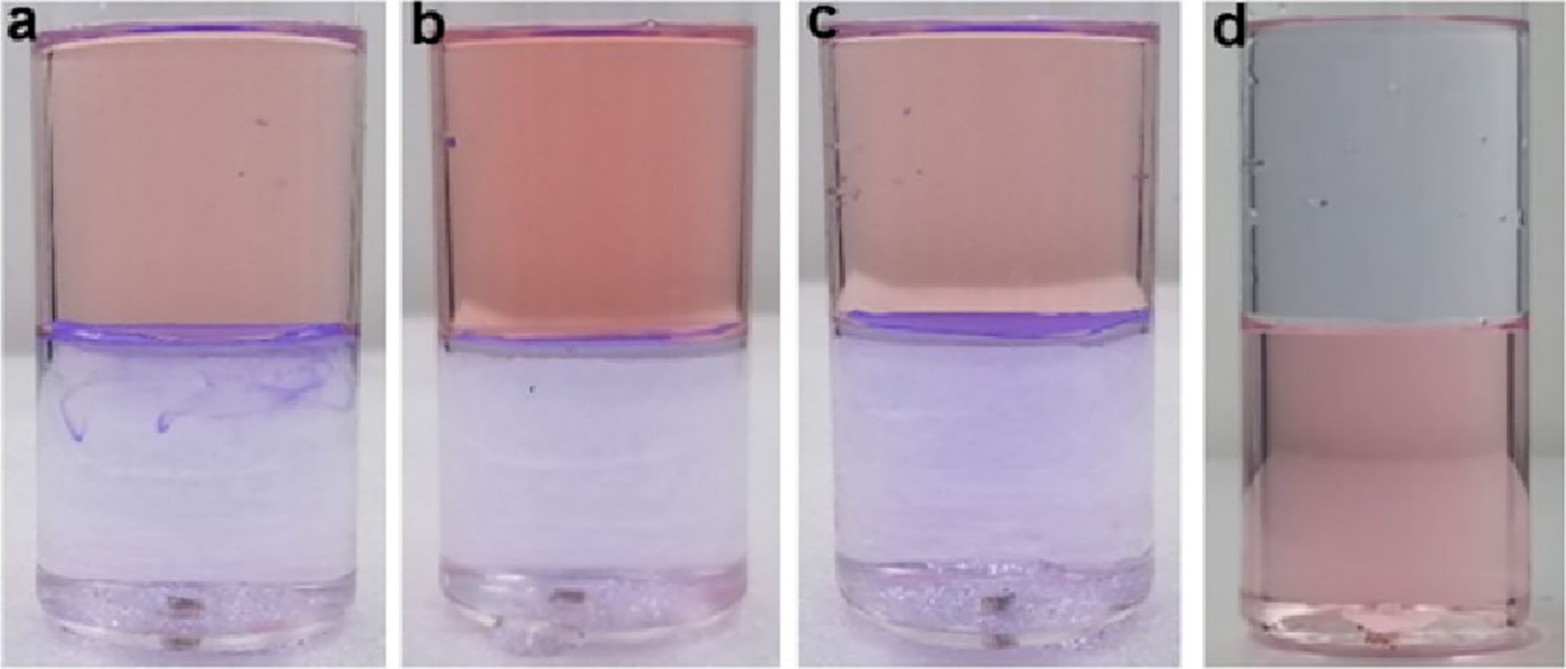
The water-infused CA (pore size: 0.22 μm) membrane can float at the hexane–water (**a**), heptane–water (**b**), and cyclohexane–water (**c**) interface. The CCl_4_-infused CA (pore size: 0.22 μm) membrane can float at the water-CCl_4_ interface (**d**).

The CA membrane remained stably floating at the petroleum ether/water interface even after rocking the mixture several times. This strong floating capability was also observed at other immiscible oil/water interfaces, including hexane/water, heptane/water, and cyclohexane/water (Fig. 8a–c). Similarly, a CCl_4_-infused CA membrane stably floated at a water/CCl_4_ interface (Fig. 8d).

### Ultrafast oil/water separation

**Figure 9:**
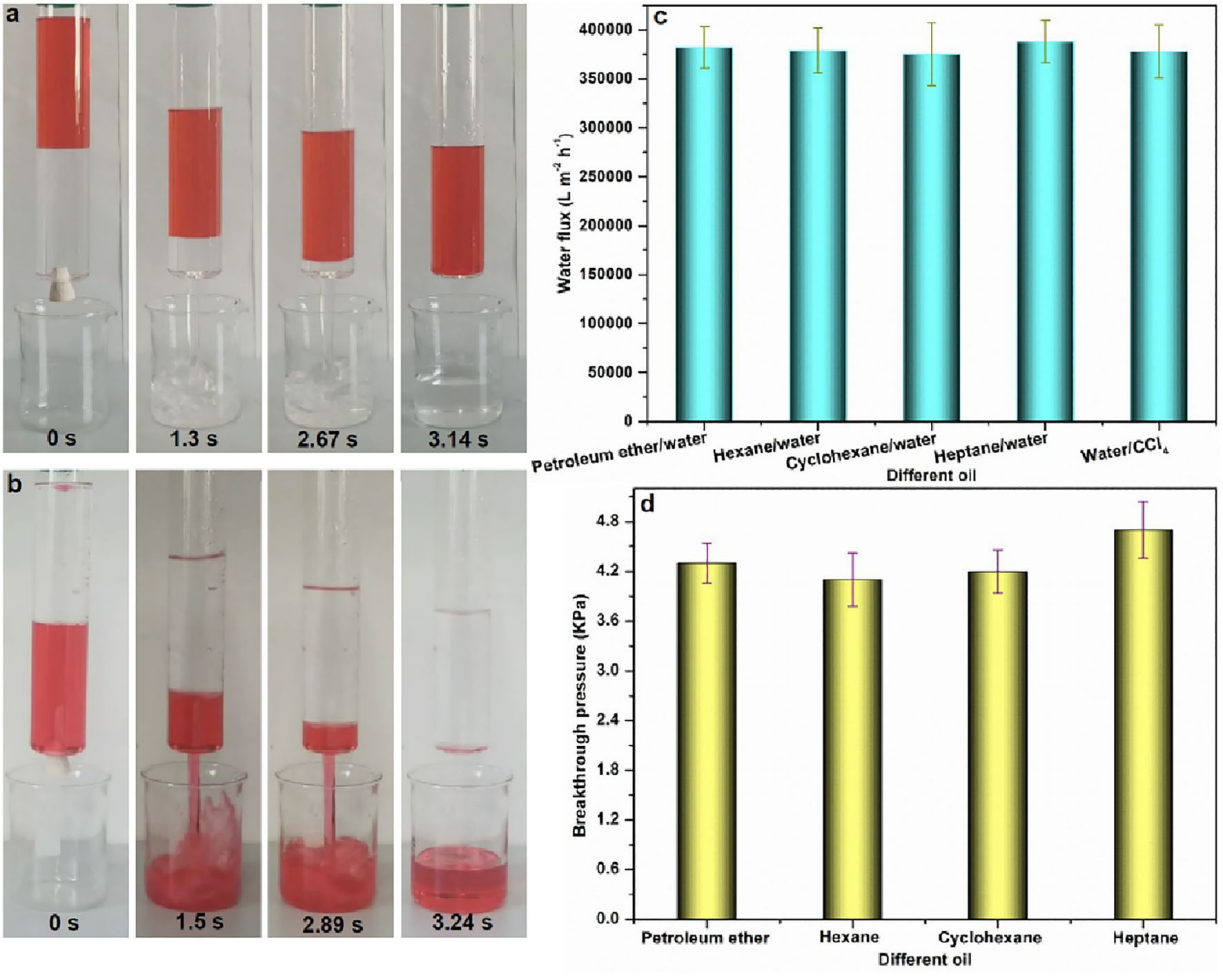
Oil/water separation results of LIIFPM system. (**a**) Water pre-wetted CA (pore size: 0.22 μm) separates petroleum ether (dyed by Oil Red)-water mixture (50–50 mL), (**b**) CCl_4_ pre-wetted CA (pore size: 0.22 μm) separates water-CCl_4_ (dyed by Oil Red) mixture (50–50 mL), (**c**) The flux results of LIIFPM system, (**d**) The breakthrough pressure of the water pre-wetted CA membrane (pore size: 0.22 μm).

Based on the aforementioned findings, we devised a novel water-infused porous membrane for the ultrafast separation of oil/water systems. Employing the LIIFPM method, we used a glass separation tube equipped with a hole (diameter: 1 cm) at its base for water treatment. The glass tube was filled with a mixture of petroleum ether (100 mL) and water (100 mL), and a water-infused CA membrane was placed in the tube (Fig. 9a). Upon opening the rubber plug at the bottom of the tube to initiate separation, the water rapidly flowed out of the tube, while the flow of the petroleum ether was stopped by the water-infused CA membrane.

The recorded water flux was approximately 370,000 L m^−2^ h^−1^ (Fig. 9b), which is several orders of magnitude higher than that observed in conventional filtration systems^39,40,48^. Because the water (lower-layer liquid) does not pass through the porous membrane, the water flux is not controlled by the pore size of the membrane. That is, the performance of the LIIFPM is independent of the membrane pore size (Fig. 9a).

Furthermore, the water-infused CA membrane was able to separate diverse immiscible oil/water mixtures, including heptane/water, hexane/water, and cyclohexane/water mixtures, with exceptional separation performance (Fig. 9c). Similarly, a CCl_4_-infused CA membrane was able to separate a water/CCl_4_ mixture, with a flux of above 370,000 L m^−2^ h^−1^ (Fig. 9a and c).

To examine the separation capacity of the water-infused CA membrane (pore size: 0.22 μm), we measured the breakthrough pressures of different oils, including petroleum ether, hexane, cyclohexane, and soybean oil, as they flowed through the porous membrane. This indicated the maximum pressure of liquids that the water-infused CA membrane could support. Owing to the small pore size of the water-infused CA membrane, the average breakthrough pressures for all the selected oils exceeded 4.0 kPa (Fig. 9d). The oils were unable to flow through the membranes below their breakthrough pressure, demonstrating the superior oil/water separation capability of this membrane.

## Conclusion

We propose a distinct LIIFPM method for ultrafast immiscible oil/water separation. The superamphiphilic porous membrane functions similarly to a reservoir, in that it is simply filled by a wettable liquid to form a defect-free liquid film that repels immiscible liquids. Owing to its superamphiphilicity, the liquid-infused porous membrane floats stably at oil/water interfaces, acting as a movable barrier for immiscible oil/water separation. The LIIFPM system, independent of the membrane pore size, can efficiently separate a variety of oily wastewaters, including petroleum ether/water, hexane/water, cyclohexane/water, heptane/water, and water/CCl_4_. Furthermore, the high breakthrough pressure demonstrates that the LIIFPM has a high separation capacity.

## Data Availability

All data generated or analysed during this study are included in this published article.
